# Technical Note: A Homemade Light Shutter to Shed Light on Electron–Hole Recombination of TiO_2_ and BiVO_4_ during Water Photoelectrooxidation

**DOI:** 10.1021/acsomega.5c00546

**Published:** 2025-05-22

**Authors:** Silvio M. Mazarin, Willy B. Kira, Daniel F. da Costa-Filho, Cinthia R. Zanata, Heberton Wender, Cauê A. Martins

**Affiliations:** Institute of Physics, 54534 Universidade Federal de Mato Grosso do Sul, CP 549, Campo Grande, Mato Grosso do Sul 79070-900, Brazil

## Abstract

Photoelectrochemical (PEC) water oxidation is a promising strategy for renewable energy conversion, but the intricate dynamics of electron–hole recombination in photoanodes require advanced methodologies for precise investigation. While essential for controlled illumination in photocatalysis, existing light shutters are often expensive, inflexible, or challenging to customize for specific experiments. To address this gap, we developed a low-cost, Arduino-controlled mechanical light shutter designed for versatile and precise control of light exposure during photoelectrochemical studies. The system was tested using TiO_2_ and BiVO_4_ photoanodes for PEC water oxidation. The light shutter was assembled using off-the-shelf components and programmed for variable cutoff times ranging from 0.1 to 50 s. The results revealed distinct transient photocurrent spikes in BiVO_4_ at low scan rates (<10 mV/s), also observed under potentiostatic (low and high bias) conditions, attributed to significant electron–hole recombination, while TiO_2_ exhibited lower recombination effects due to its faster water oxidation kinetics, as expected. Compared with existing designs, our light shutter stands out for its simplicity, cost-effectiveness, and ease of integration, providing precise and reliable light modulation without requiring complex fabrication, specialized components, or advanced programming skills. The findings highlight the ability of the developed light shutter to elucidate recombination dynamics by accurately modulating light exposure. This tool provides an accessible and effective alternative for exploring photocatalytic materials, offering insights into their charge carrier behavior and advancing the development of efficient photoanodes for solar-driven water splitting.

## Graphical abstract

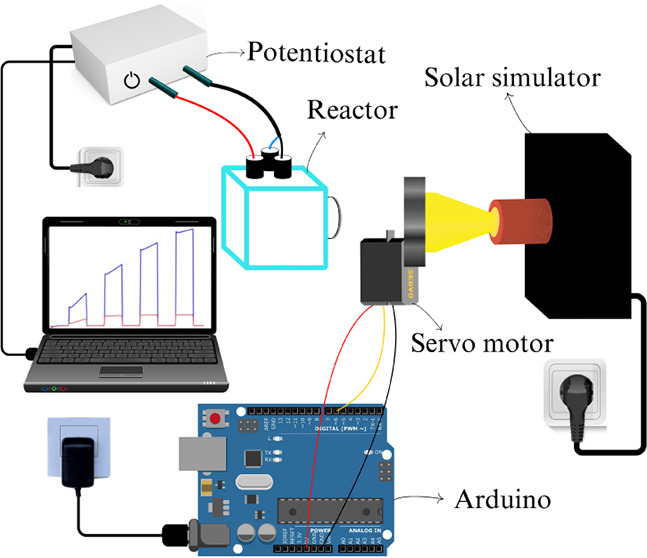

## 1 Introduction

Photoelectrochemical (PEC) and photocatalytic systems have garnered considerable interest for their pivotal roles in addressing environmental and energy challenges, including pollutant degradation and water splitting.^1,2^ These systems involve complex processes such as charge accumulation, recombination, and transfer, which are highly sensitive to the precision of light exposure. Controlled illumination, often achieved through light-source shutters, is essential for studying transient photoinduced phenomena and advancing PEC technologies and solar cell research.^3–5^

Transient phenomena, such as electron–hole recombination, are critical in shaping the efficiency of photocatalysts such as TiO_2_ and BiVO_4_. Understanding and precisely controlling light exposure—its intensity, wavelength, and duration—are vital for investigating these processes, as charge carrier dynamics under illumination are highly light-dependent. Despite their advantages, TiO_2_, celebrated for its stability and strong oxidative properties, and BiVO_4_, valued for its visible-light activity, both face significant performance limitations due to recombination losses.^6,7^ Studying the effects of light exposure on these dynamics provides essential insights into when and how recombination occurs. This knowledge guides the development of strategies such as doping, surface modification, and heterojunction engineering, which extend charge carrier lifetimes and improve photocatalytic efficiency.^8–10^ By mastering the interplay between light exposure and recombination, researchers can optimize TiO_2_ and BiVO_4_ for advanced energy and environmental applications.

Precise light control is necessary to regulate irradiation periods, ensuring a stable reaction environment and enhancing reproducibility across experiments, especially for short-term experiments.^11^ Without precise light control, results may be unreliable, particularly when using light-sensitive catalysts and fast kinetics, where inconsistent light intensity or duration can lead to variability in reaction yields.^12^ Commercial shutters are integral to these applications, allowing for adjustable light exposure. Among the commercial ones, there are various choices from different companies.^13–15^

However, commercially available light shutters often present challenges in terms of cost, complexity, and limited adaptability. Many high-precision shutters are excessively expensive,^13,15^ designed for broad applications but lack the customization required for specific photocatalysis or laser experiments.^16,17^ Additionally, these shutters often require extensive calibration and sophisticated software, which can be challenging in resource-limited settings.^13^

To overcome these commercial light shutter challenges, various researchers have designed different types of shutters according to their specific needs.^18^ Light shutters can be categorized into several types, each with distinct performance characteristics. Mechanical shutters, for instance, offer high durability and precise timing but suffer from slower response times and mechanical wear.^19,20^ Electronic shutters, such as liquid crystal or electro-optic shutters, provide faster switching times but can be more expensive and prone to overheating; they also partially obstruct light. Each shutter type involves trade-offs between speed, cost, and robustness, which may limit their suitability for dynamic photocatalysis applications requiring rapid on–off cycles and fine adjustments.^20^ Stability and vibration control are additional concerns in many experimental setups, as these factors can impact shutter performance.^19^ H. Zhang et al. developed a mechanical laser shutter powered by a DC motor to drive a 3D-printed blade, achieving a switching speed of 1.22 m/s with exceptional reliability (1 ms activation delay) and precision (10 μs jitter), demonstrating durability over 10^8^ cycles.^21^ In the pursuit of adaptable shutters for experimental use, M. Bauer et al. designed and implemented a mechanical shutter based on a bending piezo-actuator, featuring an optimized aperture diameter of up to 2 mm.^16^

Recent advances in open-source hardware, such as Arduino microcontrollers, offer a promising alternative to commercial shutters, allowing for custom, low-cost solutions with a high degree of flexibility.^22^ Arduino-based systems can be programmed for precise control over light exposure duration, providing advantages in scalability, accessibility, and adaptability to specific experimental needs. With Arduino technology, it is feasible to achieve comparable or improved shutter performance at a fraction of the cost of commercial models. For instance, Mathias S. Fischer and Martin C. Fischer developed an open-source, low-cost optical shutter system using Arduino-based controllers and ubiquitous actuators (servo motors or solenoids).^22^ Their design is adaptable, supporting multiple control options, 3D-printable components, and customizable interfaces for various optical experiments.^22^ However, its structure and software interface may pose challenges for those unfamiliar with programming and system assembly.

In this work, we aim to design and implement a simple, low-cost light shutter using Arduino technology to enhance accessibility to precise light control in photocatalytic research. We developed a tailored shutter system with performance characteristics compatible with photoelectrocatalysis, such as response time and synchronization capabilities, while reducing operational costs in laboratory experiments. We provide all resources, from physical parts to software, to make this material available for other researchers. As a proof of concept, we used this new light shutter to investigate PEC water oxidation on TiO_2_ and BiVO_4_ photoanodes.

## 2 Experimental Section

### 2.1 Assembly of the Electronic Device

The system used to control light incidence or dark conditions (the light shutter) on the photoelectrode consists of an electronic logic module and peripheral components. Figure 1 illustrates the materials used for the assembly of the device. The Arduino UNO electronic module (Figure 1A,B) features a processor, memory, and analog and digital input/output buses. It is responsible for operating the peripheral devices and executing preprogrammed commands stored in its internal memory. These commands are programmed through a custom coding/algorithm specifically developed for this task (Supporting Information Section I). The algorithm is uploaded to the module using the free Arduino-specific interface software, IDE (Integrated Development Environment) version 2.3.3, and is transferred to a servo motor (Figure 1C) via a USB connection cable (Figure 1D). The project diagram is depicted in Figure 1B. The servo motor is connected to a 2 cm × 10 cm plastic plate, which can be wrapped with aluminum foil for photocatalytic or PEC applications.

**Figure 1:**
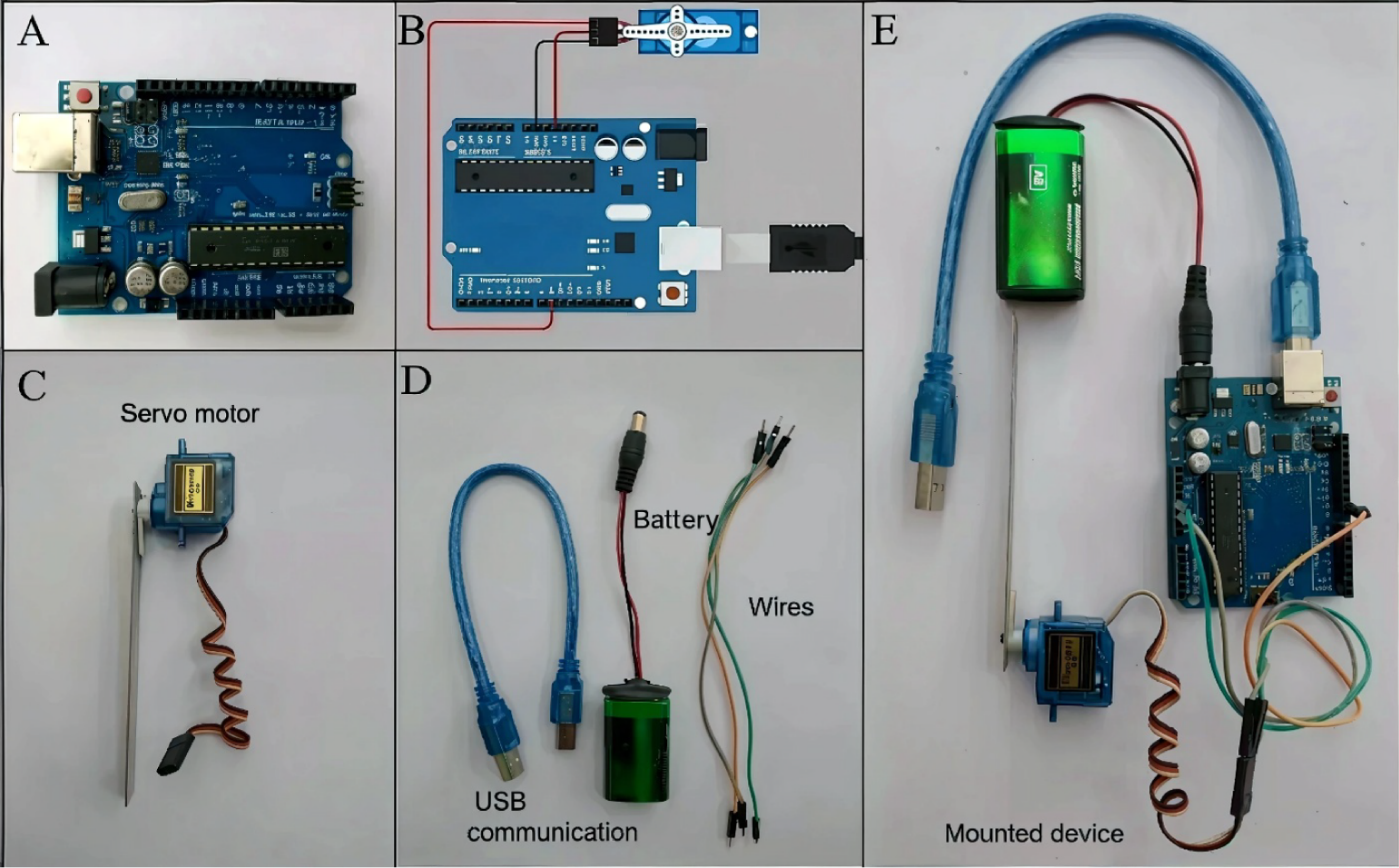
(A) Arduino UNO board. (B) Schematic of the electronic circuit designed using Tinkercad online software. (C) Servo motor. (D) Communication and connection accessories. (E) Fully assembled circuit, ready for operation.

Electrical connections are made using wires, and the device is powered by either a conventional 9 V battery or a USB when connected to a computer (Figure 1D). Figure 1E shows the fully assembled device, which operates by moving the servo motor from 0° to 90° and back from 90° to 0°, as dictated by the time intervals programmed into the algorithm. This routine continues in a loop until the user interrupts the cycle to input new time parameters into the algorithm.

The light or dark time of the shutter, referred to here as the “cutoff time,″ was programmed by modifying the value of the “delay” command in the code, expressed in milliseconds. This adjustment applies to both the 0° and 90° positions. After setting the desired value, the “upload” command was executed to record the new operating time. Supporting Information Section I shows a step-by-step building of the light shutter, featuring photos and screenshots (Figures S1–S6) and the coding for copy and paste.

### 2.2 Synthesis of TiO_2_/Ti and BiVO_4_/FTO

TiO_2_ nanotubes (NTs) were obtained via a single-step anodization process^23^ in ethylene glycol (EG) with 0.5 wt % NH_4_F and 2 vol % water. Titanium plates were cut into 1 cm × 2 cm dimensions, cleaned using an ultrasonic bath with deionized water, followed by ethanol, and subsequently dried with an Ar gas stream. The anodization process employed a 1 cm × 1 cm Ti substrate, delimited by Kapton tape, as the anode and a 2 cm × 1 cm Cu plate as the cathode. Anodization was carried out by applying a potential of 36 V for 30 min, keeping the setup in an ultrasonic bath. The anodized TiO_2_ NTs were rinsed with deionized water, dried with an Ar gas stream, and subjected to thermal treatment at 450 °C for 1 h in a muffle furnace to achieve the anatase crystalline phase.

BiVO_4_ photoanodes were synthesized using a two-step deposition method based on the procedure described by Kang et al.^24^ with minor modifications. The first step involved the electrodeposition of a metallic Bi layer using a three-electrode electrochemical cell. The working electrode consisted of FTO (1 × 1 cm), the reference electrode was Ag/AgCl (3 mol L^–1^ KCl), and the counter electrode was Pt (1 × 2 cm). The electrolyte was an ethylene glycol solution containing 20 mM Bi­(NO_3_)_3_·5H_2_O. A charge of 0.32 C cm^–2^ was applied at E = −1.8 V vs. Ag/AgCl. After deposition, the Bi film was carefully rinsed with ethanol and dried with an air stream. The second step involved converting the Bi film to BiVO_4_ by depositing 100 μL cm^–2^ of a 0.2 mol L^–1^ solution of VO_2_(acac) in dimethyl sulfoxide (DMSO) onto the Bi-coated electrode surface. After natural drying, the film was heated at 450 °C for 2 h in a muffle furnace at a heating rate of 2 °C min^–1^. Residual V_2_O_5_ was removed by immersing the electrode in a 1 mol L^–1^ NaOH solution under stirring for 30 min. Morphology of TiO_2_ NTs and BiVO_4_ photoanodes was investigated by scanning electron microscopy (SEM), and structural characterization was performed by Raman spectroscopy.

### 2.3 Electrochemical Measurements

To investigate the capability of the light shutter, PEC water oxidation was chosen as a model reaction. Electrochemical measurements were conducted as depicted in the schematic shown in Figure 2A. The Arduino-based light shutter controls the cutoff time, exposing and protecting the photoanode from simulated sunlight. A three-electrode cell (Figure 2B) made of Teflon was employed, using a Pt plate as the counter electrode and an Ag/AgCl (3 M KCl) electrode as the reference. The TiO_2_/Ti and BiVO_4_/FTO photoelectrodes, with a submerged area of 1 cm^2^ were used as working electrodes, ensuring current normalization. The cell was equipped with a quartz window to enable frontal light exposure to the semiconductor surface (Figure 2B).

**Figure 2:**
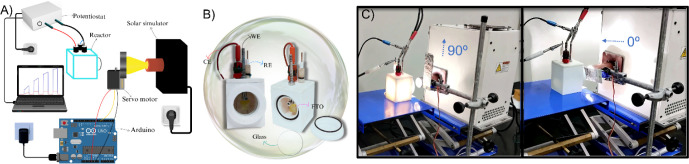
(A) Schematic representation of the experimental setup for Arduino-controlled measurements. (B) Detailed view of the reactor used for half-cell measurements under light irradiation, highlighting the electrode configuration. (C) Illustration of device operation under illuminated and nonilluminated conditions. Photos were captured by the authors.

The SEM images confirmed the nanotube morphology of the TiO_2_ film, with a film thickness of ∼2.3 μm (Figure S7). The BiVO_4_ demonstrated the formation of worm-like nanoporous particles (Figure S7). Raman spectra (not shown) confirmed the formation of the anatase phase for TiO_2_ NTs and monoclinic scheelite for BiVO_4_, as expected for the annealing temperature interval range.^25–27^

Figure 2C shows the device operation with and without light exposure. All photocurrent measurements were performed using a Gamry Interface5000 potentiostat/galvanostat, applying potentials ranging from −0.5 to 0.8 V vs. Ag/AgCl in an oxygen-free 0.5 mol L^–1^ sodium borate buffer solution (BBS, pH 9.3). The potentials were converted to the reversible hydrogen electrode (RHE) scale using the Nernst equation (*E* = *E*° + 0.0591 pH). Current responses were recorded in the potential range −0.5 to 0.8 V *vs*. Ag/AgCl at scan rates of 2 mV s^–1^ and 10 mV s^–1^ under 200 mW cm^–2^ light intensity provided by a solar simulator (Abet Technologies, 150 W Xe arc lamp) equipped with an AM 1.5G filter. The photocurrents were normalized to the semiconductor geometric area. The light intensity was calibrated to 200 mW cm^–2^ using a silicon reference cell. These conditions allowed precise evaluation of the photoelectrodes’ behavior and the performance of the light shutter under controlled PEC conditions.

The performance of the light shutter was investigated by monitoring the PEC water oxidation with different cutoff times. Both catalysts were tested under light cutoff times of 1, 5, 10, and 15 s at a scan rate of 10 mV s^–1^, and for 20, 25, 40, and 50 s at a scan rate of 2 mV s^–1^.

## 3 Results and Discussion

The light shutter operates stably with 100 ms as the minimum cutoff time, namely the waiting time in light or dark conditions . Supplementary Movie M1 illustrates the light shutter operating from 150 ms up to 10 s of symmetrical cutoff time, with equal time to move from 0° to 90° and back to 0°. The servo’s movement time remained consistent and very close to 0.15 s regardless of the waiting time, as indicated by the manufacturer. Figure S8 shows that a reliable on–off light shutter interval for beam modulation using a photodiode and an oscilloscope is 80 ms. Lower intervals reveal irregularities. However, a semiconductor catalyst functions properly at intervals of 150 ms due to intricate photoexcitation kinetics. After confirming the stability of the device, we investigated the PEC water oxidation using the developed system and TiO_2_ NTs or BiVO_4_ photoanodes.

The linear sweep voltammograms presented in Figure 3 compare the performance of BiVO_4_ and TiO_2_ NTs photoelectrodes under different shuttered light conditions at a 10 mV s^–1^ scan rate. The BiVO_4_ photoelectrode consistently exhibits significantly higher photocurrent densities compared to TiO_2_ across all light time intervals. This superior performance highlights the efficient light absorption and charge separation capabilities of BiVO_4_, making it a more suitable candidate for PEC water oxidation under simulated solar light conditions. The obvious difference in PEC activity comes from the well-known visible light response of BiVO_4_, as compared to TiO_2_, which is photoactive only under UV light.^25,26^

**Figure 3:**
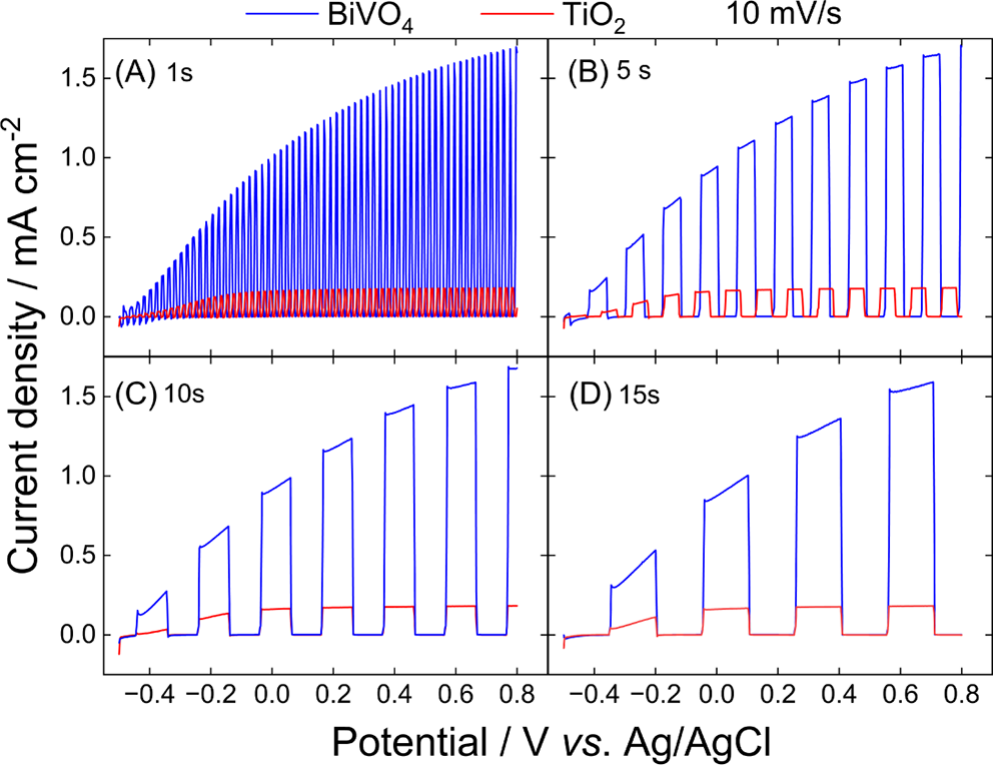
Linear sweep voltammograms of BiVO_4_/FTO and TiO_2_/Ti photoelectrodes recorded in an H_3_BO_3_ buffer solution (pH 9.3) saturated with Ar at a scan rate of 0.01 V s^–1^. Measurements were conducted with varying light cutoff intervals: (A) 1 s, (B) 5 s, (C) 10 s, and (D) 15 s. The light intensity was set to 200 mW cm^–2^. Potentials were recorded against an Ag/AgCl reference electrode, and light modulation timing was controlled by the Arduino-based light shutter system.

The effect of light exposure time intervals is particularly evident in the BiVO_4_ electrode. At shorter intervals (1 s), the photocurrent displays pronounced oscillations, reflecting the electrode’s rapid and reproducible response to changes in light-on times (Figure 3A). As the light interval increases to 5 (Figure 3B), 10 (Figure 3C), and 15 s (Figure 3D), the photocurrents stabilize into well-defined patterns. The TiO_2_ NTs photoanode can reach a quasi-steady-state regime at 0 V vs. Ag/AgCl during illumination. This plateau is due to slow surface reaction kinetics for water oxidation; therefore, increasing the bias further does not significantly increase the current. BiVO_4_ does not achieve steady photocurrent density plateaus, demonstrating its ability to further increase the photocurrent with increasing bias potential.

Another critical point is obtaining the onset potential using LSV curves with intermittent light conditions, as it requires high frequencies (low cutoff times) for precise determination, justifying the use of automatic light shutters. As can be seen, the 1 s resolution of light cutoff is the better choice for determining the onset potentials. The BiVO_4_ photoelectrode achieves a lower onset potential (below −0.5 V, outside the range of measurement) compared to TiO_2_ (∼−0.48 V), suggesting that BiVO_4_ requires less external bias to initiate the water oxidation reaction under solar light illumination. This characteristic is crucial for developing efficient PEC systems, as it reduces energy input requirements while maintaining high photocurrent densities.

The results in Figure 4 provide a deeper understanding of the PEC water oxidation kinetics over BiVO_4_ and TiO_2_ photoelectrodes, as the experiments were performed at a lower scan rate of 2 mV s^–1^ compared to the results in Figure 3 (10 mV s^–1^). By slowing the scan rate, it becomes possible to observe additional dynamic processes through “spikes” formation when the electrode is exposed to light, especially for BiVO_4_. These spikes started to manifest in BiVO_4_ at 10 mV s^–1^ but with a clearer signal at 2 mV s^–1^. This strong effect at a lower voltage scan rate may arise from instantaneous charge accumulation followed by fast electron–hole recombination, revealing the slow interfacial hole transfer kinetics of BiVO_4_ during water oxidation.^28^ It is less apparent at higher scan rates and reveals important differences in the PEC activity and charge carrier dynamics of the two photoanodes.

**Figure 4:**
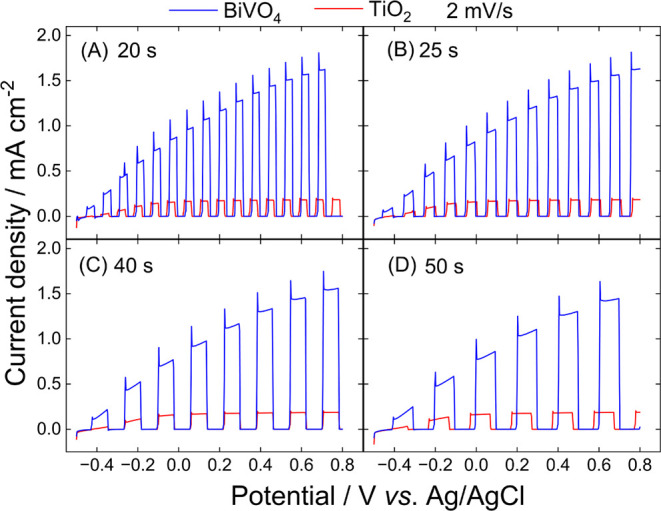
Linear sweep voltammograms of BiVO_4_/FTO and TiO_2_/Ti photoelectrodes recorded in an H_3_BO_3_ buffer solution (pH 9.3) saturated with Ar at a scan rate of 2 mV s^–1^. Measurements were conducted with varying light cutoff intervals: (A) 20 s, (B) 25, (C) 40, and (D) 50 s. The light intensity was set to 200 mW cm^–2^. Potentials were recorded against a Ag/AgCl reference electrode, and light exposure timing was controlled by the Arduino-based light shutter system.

Distinct current “spikes” are visible in the BiVO_4_ voltammograms during different light modulation intervals (Figure 4A-D). When light is suddenly turned on, a certain number of photogenerated carriers are initially available, resulting in a spike in photocurrent. However, due to inefficient charge injection into the electrolyte and/or the presence of surface traps on the photoanodes, many holes recombine at the photoelectrode surface rather than participating in the water oxidation reaction, thereby decreasing the photocurrent to a steady-state value.^28–30^ At a lower scan rate, the system has more time to approach equilibrium conditions, allowing the charge recombination dynamics to manifest clearly. Figure 4C and 4D show that LSV at 2 mV s^–1^ reveals this phenomenon even at high cutoff times, such as 40 and 50 s, as seen in details in Figures S9 and S10. In contrast, these spikes are much less pronounced in the TiO_2_ voltammograms, indicating that, although showing limited PEC activity, TiO_2_ has more efficient interfacial hole transfer kinetics, or fewer surface traps, compared to BiVO_4_. Figures S11–S12 present comparisons at different cutoff times under a constant scan rate, illustrating that even TiO_2_ experiences electron–hole recombination, although with lower current magnitudes. This behavior is observed at low scan rates and remains consistent regardless of the cutoff time (Figure S11).

As this phenomenon can be better visualized at a constant voltage bias, we performed chronoamperometry at 0.2 and 0.6 V, as shown in Figures S13 and S14, respectively. We chose two bias conditions to evaluate the band bending effect on spike formation. At low bias (0.2 V vs. Ag/AgCl, Figure S13), both TiO_2_ NTs and BiVO_4_ photoanodes exhibited spike formation for all investigated cutoff times (1, 5, 10, and 15 s). After charge separation at low bias, the spikes in photocurrent arise from the slow kinetics of the water oxidation reaction that cannot sustain a high rate of hole consumption, causing the photocurrent to drop back. At higher bias (0.6 V vs. Ag/AgCl, Figure S14); however, the photocurrent from TiO_2_ NTs reaches a plateau (steady state) under light conditions, while spikes appear to increase for the BiVO_4_ photoanode, revealing different behavior for the two semiconductor photoanodes. This observation aligns with findings by Zhu et al., who identified surface recombination in BiVO_4_ as a limiting factor in its photoelectrochemical performance.^31^

It is well known that a built-in potential arises at the semiconductor/electrolyte interface from the difference in the Fermi levels of the semiconductor and the electrolyte at equilibrium, creating a band bending close to the interface.^32^ Additionally, when an anodic bias is applied to an n-type semiconductor, such as BiVO_4_ and TiO_2_, it increases band bending, widening the depletion region and enhancing the separation of photogenerated electron–hole pairs.^33^ Moreover, monoclinic BiVO_4_ has a narrower band gap, a more positive conduction band, and a lower carrier density than anatase TiO_2_,^1,7^ leading to weaker initial band bending at the semiconductor/electrolyte interface.^32^ As a result, higher applied biases are required for BiVO_4_ photoanodes to sufficiently widen the depletion region and equilibrate charge separation and charge injection to the electrolyte compared to TiO_2_.

These observations further highlight the superior ability of TiO_2_ for charge injection at the semiconductor/electrolyte interface during PEC water oxidation, although it has poor overall PEC activity compared to BiVO_4_ due to its limited absorption of solar photons. However, the visible light absorption and the ability of BiVO_4_ to recover its photocurrent during subsequent light-on intervals indicate efficient regeneration of active carriers, which contributes to its overall superior performance.

The suppression of the spikes is easily observed by adding Na_2_SO_3_ to the electrolyte solutions, as shown in Figure S15. Sodium sulfite is an efficient hole scavenger that reacts with photogenerated holes at the semiconductor/electrolyte interface, converting them into sulfate. Moreover, sulfite oxidation exhibits kinetics significantly faster than those of the oxygen evolution reaction (OER), effectively minimizing hole accumulation at the semiconductor surface. This rapid reaction suppresses transient charge recombination or surface trapping events, thereby preventing spikes in the photocurrent, as expected and in line with the above discussion.

Calculations of charge transfer efficiencies (η_trans_) were made using the formula $\eta_{\mathrm{trans}}=\frac{J_{\mathrm{ss}}}{J_{\mathrm{inst}}}$ , where *J*_ss_ is the steady-state current density, and *J*_inst_ is the instantaneous maximum current density at the ″light-on″ state (Table S1). All values were based on Figure 4, averaging voltammogram spikes at 2 mV s^–1^ from −0.2 to 0.2 V for 20, 25, 40, and 50 s of light cutoff intervals. In this case, as *J*_ss_ approaches *J*_inst_ the *η*_trans_ tends to unity. This analysis during potentiostatic and transient light conditions offers valuable insights into the electron–hole recombination dynamics of BiVO_4_ and TiO_2_ photoelectrodes. Notably, *η*_trans_ remains consistent at about 86% and 82% for TiO_2_ NTs and BiVO_4_, respectively, regardless of the light cutoff time, indicating that the observed current spikes are intrinsic to the materials’ properties and are not influenced by the mechanical operation of the light shutter. Moreover, TiO_2_ exhibits a more subdued transient response, with a slightly higher *η*_trans_ (Table S1) and relatively less impact from surface recombination. In contrast, BiVO_4_ suffers from significant surface recombination, primarily due to its slower water oxidation rate, which leads to the accumulation of surface holes—unlike TiO_2_, where rapid water oxidation effectively reduces such recombination effects.^31^

Thus, although BiVO_4_ exhibits higher photocurrents for PEC water oxidation due to its broad wavelength absorption, its slow water oxidation kinetics significantly enhance electron–hole recombination (Figures S13–S14).

The consistency of the *η*_trans_ across different light cutoff times emphasizes the reliability of the Arduino-controlled light shutter system in probing the kinetics of charge accumulation, electron–hole recombination, and charge transfer, all at the semiconductor/electrolyte interface. By providing precise control over illumination intervals, this system enables the differentiation of intrinsic material properties from external factors, facilitating a clearer understanding of the recombination processes in various photoelectrodes.

The comparative analysis of transient photocurrent responses reveals that BiVO_4_, despite its higher initial photocurrent generation, suffers from significant recombination losses, whereas TiO_2_ demonstrates a more gradual photocurrent response, indicative of lower recombination rates. The use of a controlled light shutter system proves effective in elucidating these dynamics, offering a valuable tool for studying electron–hole recombination kinetics in photoelectrochemical materials.

In summary, the light shutter developed in this study offers a practical and cost-effective alternative to existing designs, overcoming several limitations reported in the literature. Previous works have explored various approaches, including piezoelectric actuators,^16^ DC motors,^21^ and electromechanical systems,^22^ each with distinct trade-offs in complexity, precision, and applicability. All of these works are important and contribute to knowledge. For instance, the piezoelectric-based system described by Bauer et al. achieved a rapid response time of approximately 120 μs;^16^ however, its implementation required specialized components, signal generators, and oscilloscope-based characterization, making it less accessible for general laboratory use. Similarly, the DC motor-driven shutter presented by Zhang et al. incorporated 3D-printed components and required a custom control circuit, which, although effective, adds complexity and may not be practical for researchers without experience in electronics prototyping.^21^ The open-source design proposed by Fischer and Fischer provided an Arduino-controlled shutter with detailed assembly instructions, yet its use of 3D-printed parts and additional control modules increased the cost and complexity beyond what is necessary for standard photocatalytic applications.^22^ In contrast, our light shutter is distinguished by its simplicity, affordability, and ease of integration into existing experimental setups. Unlike designs that require custom fabrication or specialized components, our system utilizes widely available materials and requires minimal assembly, making it accessible to a broader range of researchers. The calculated light obstruction time of approximately 9.55 ms (Supporting Information Section I) is comparable to or better than some reported designs, ensuring reliable modulation of illumination for transient photocurrent studies. Additionally, many of the previous studies focused on validating their shutters using laser beams detected by photodiodes without demonstrating applicability in photocatalysis. Our approach not only provides an effective light modulation tool but also directly applies it to photoelectrochemical water oxidation, revealing important insights into charge carrier dynamics. This integration of a practical, low-cost device with a relevant scientific application highlights the novelty of our work, offering a valuable contribution to the field of photoelectrocatalysis.

## 4 Conclusions

This study introduced a low-cost, Arduino-based light shutter as a practical and efficient tool for controlling light exposure during photoelectrochemical experiments. By addressing the limitations of commercial shutters, this system provides a versatile alternative for researchers, allowing precise modulation of light intensity and exposure times. The developed light shutter offers a highly accessible and adaptable solution for photoelectrochemical studies, combining low cost and straightforward assembly with performance comparable to that of more complex and expensive commercial and research-grade systems. The device was tested in the context of PEC water oxidation experiments using TiO_2_ and BiVO_4_ photoanodes, shedding light on their electron–hole recombination dynamics under controlled illumination.

The experiments revealed key differences in the photoelectrochemical behaviors of the two materials. BiVO_4_ demonstrated higher photocurrent densities, attributed to its broader wavelength absorption range, particularly in the visible spectrum. However, its slower water oxidation kinetics resulted in significant electron–hole recombination, as evidenced by pronounced current spikes, with an *η*_trans_ of about 82%. This behavior highlights the challenges posed by surface recombination in BiVO_4_ and shows the importance of improving its charge carrier dynamics for enhanced performance. In contrast, TiO_2_ exhibited more stable photocurrent responses with an *η*_trans_ of 86%. It is attributed to TiO_2_’s faster water oxidation kinetics, which effectively suppress surface recombination and facilitate steady-state photocurrent generation. While TiO_2_’s photocurrent densities were lower due to its limited absorption of visible light, its efficient charge separation makes it a robust candidate for UV-driven photocatalysis.

The results are as expected and emphasize the effectiveness of the Arduino-controlled light shutter in investigating intrinsic recombination dynamics. The device’s ability to maintain consistent performance across varying cutoff times demonstrates that the observed phenomena are governed by material properties rather than mechanical limitations. This reliability highlights the potential of the light shutter as a valuable tool for exploring charge carrier behavior in photoelectrochemical systems.

## Supplementary Material





## Notes

Recorded videos demonstrate how to build the light shutter and explore its potential applications . We used ChatGPT AI to enhance the grammar and language of the text­(MP4)
Algorithms utilized for the Arduino, along with experimental details for constructing the light shutter. Photoelectrochemical measurements­(PDF)
