# Supplementary material for: Technical Note: A Homemade Light Shutter to Shed Light on Electron–Hole Recombination of TiO2 and BiVO4 during Water Photoelectrooxidation
Source: ACS Omega. 2025 May 22;10(21):21529–36. doi: 10.1021/acsomega.5c00546 (PMC12138702; doi:10.1021/acsomega.5c00546)
Supplement: Supplementary file 2 [file ao5c00546_si_002.pdf]

Supplementary Information

Technical Note: A home-made light shutter to shed light on electron-hole recombination of TiO_2 and $BiVO_4$ during water photo-electrooxidation

Silvio M. Mazarin,[#] Willy B. Kira,[#] Daniel F. da Costa-Filho, Cinthia R. Zanata, Heberton Wender, Cauê A. Martins*

Institute of Physics, Universidade Federal de Mato Grosso do Sul, CP 549, 79070-900, Campo Grande, MS, Brazil.

*caue.martins@ufms.br

__Section I.__ Assembliing of the light shutter

The micro servo motor has three connection wires with standardized colors for the SG90 model (**Figure S1**), all of which need to be connected to the Arduino board as follows: the brown wire should be connected to the GND pin, the red wire to the +5V pin, and the yellow wire to pin 9 (**Figure S2**). The Arduino board must be connected to the PC via a USB cable (**Figure S3**).

To proceed, the Arduino IDE interface software should be downloaded from the official website (https://www.arduino.cc/) and installed. The user should select the latest version, currently 2.3.4 (https://www.arduino.cc/en/software). Once installed, click on the program icon (inset in **Figure 3SA**), which will open a blank screen. If anything is written on the screen, delete it and paste the provided code in its place (**Figure S4**). To establish communication with the Arduino board, navigate through the menus by selecting "Tools," then "Ports," and choose the port labeled as “COM” with “(Arduino UNO)” to enable it (**Figure S3B**). To upload the code to the Arduino, click on the upload icon (arrow) (**Figure S5**). Within two seconds, the yellow LEDs (TX and RX on the board) will flash rapidly six times, indicating that the code has been successfully uploaded, and the routine will start.

Figure S5 shows the configuration with a 500-millisecond delay for both the 0° and 90° positions. If the user wishes to adjust the movement delay, they must edit the “delay” values in the code for both positions (0° and 90°), specifying the desired time in milliseconds, as shown in the example (**Figure S6**). After any edits, the user must re-upload the code by clicking the "arrow" icon in the top menu (**Figure S6**), ensuring that the Arduino board remains connected via the USB port (**Figure S3**). The Arduino board can always be powered via the USB cable. Alternatively, it can be powered using a standard 9V battery or a common 12V DC power supply if needed. The coding may be copied from bellow and pasted in the Arduino software.

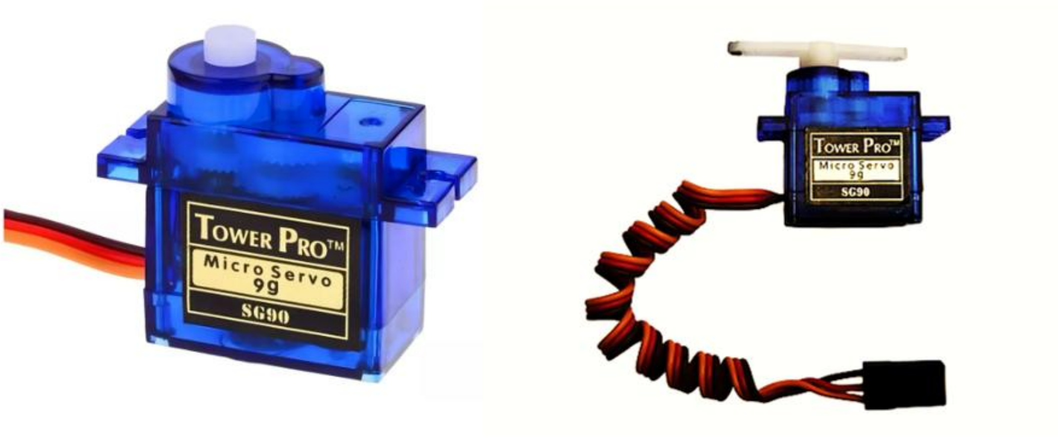

Figure S1. 9g Micro Servo Motor (SG90 model) and details of the electrical connections. Brown wire is GND, red wire is the +5V power supply, and yellow wire, the position control pin.

The SG90 servomotor has a rotation speed of 0.1 s per 60°, leading to an estimated time of 0.15 s per 90°, which is standard for 9g micro servomotors. To clarify the speed and performance of our shutter, we calculate the light obstruction time based on servomotor specifications and system geometry. Considering an aperture width of 10 mm and a distance of 100 mm from the motor axis to the light beam center, we use the following angular velocity equation:

$$\tau = \frac{360^\circ \times d}{(90^\circ/0.15) \times 2\pi L}$$

Substituting our values where *d* is the diameter of the light beam, and *L* is the distance from the motor axis to the light beam, we find:,

$$\tau = \frac{360^\circ \times 10}{(90^\circ/0.15) \times 2\pi \times 100}$$

$$\tau = 0.00955s = 9.55 \text{ ms}$$

This calculation shows that our system fully blocks or unblocks the light beam in approximately 9.55 milliseconds.

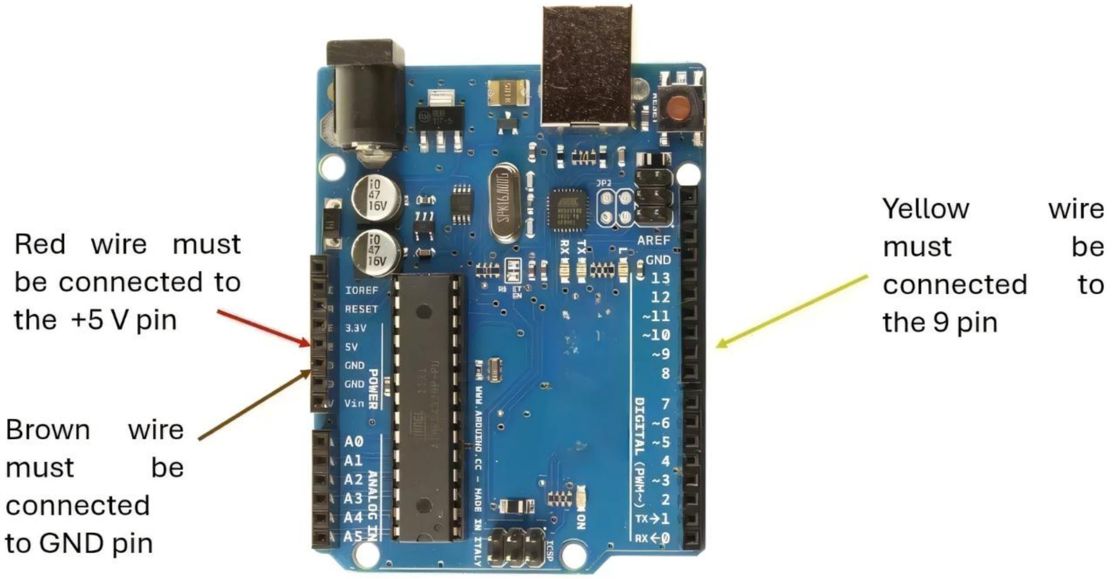

Figure S2. Connecting the servo motor to the Arduino.

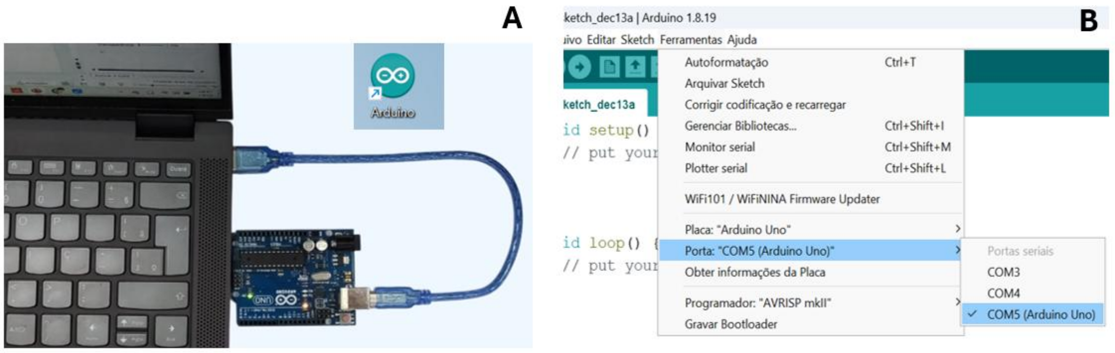

Figure S3. (a) Arduino to PC connection via USB. (b) Identification and selection of the communication port for the Arduino board.

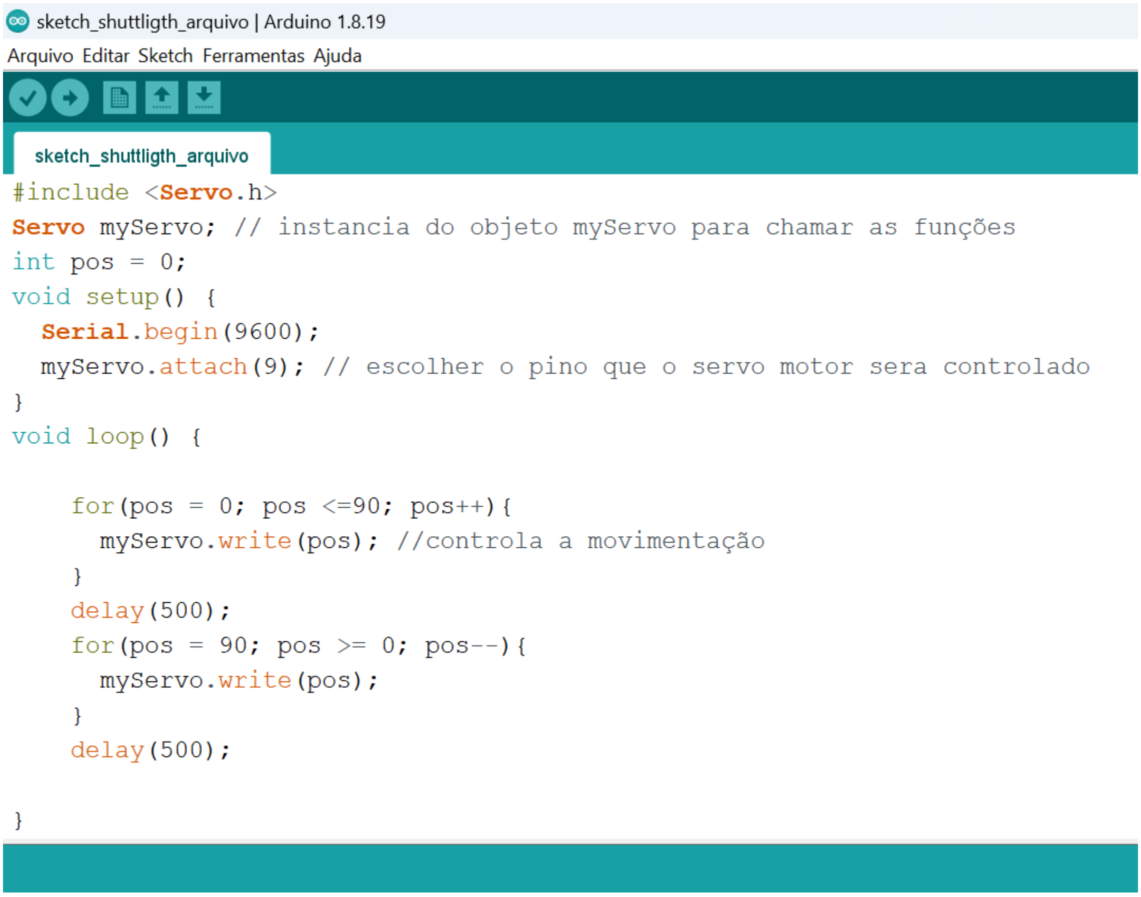

Figure S4. Arduino IDE interface screen displaying the command code.

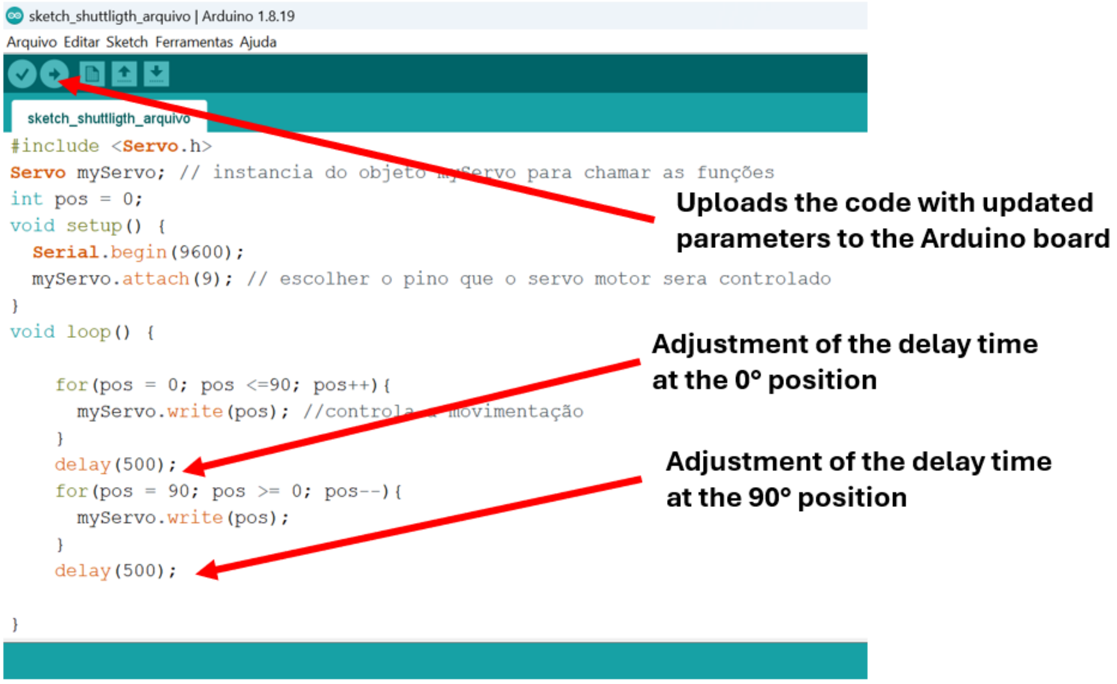

Figure S5. Arduino IDE interface screen showing the command code and instructions.

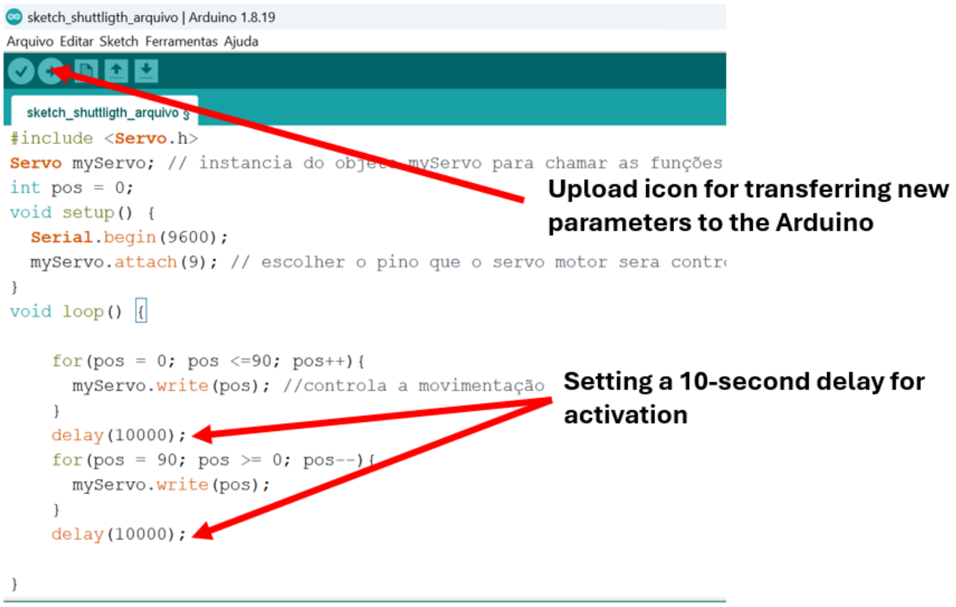

Figure S6. Configuration of activation time in milliseconds.

Coding for copy and paste into the Arduino:

```
#include <Servo.h>


Servo myServo;


void setup() {
  myServo.attach(9); // connects the servo to pin 9
}


// Function that moves the servo smoothly from 'start' to 'end'
void moveServo(int start, int end) {
  int step = (start < end) ? 1 : -1;
  for (int pos = start; pos != end + step; pos += step) {
    myServo.write(pos);
    delay(0); // time for the servo to follow the movement
  }
}


void loop() {
  moveServo(0, 10);   // upward movement
```

```
  delay(100);
  moveServo(10, 0);   // downward movement
  delay(100);
}
```

Section II. *Materials characterization*

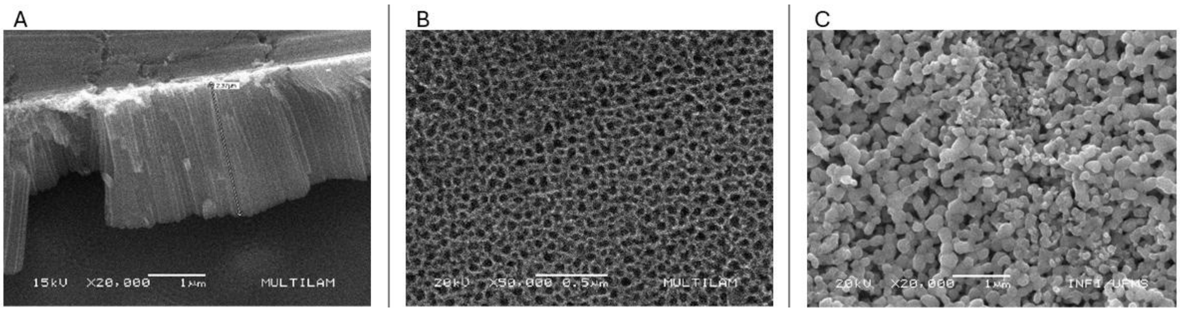

Figure S7. Representative scanning electron micrograph of the (a,b) TiO_2 nanotubes and (c) $BiVO_4$.

Section III. *Proofing the light shutter*

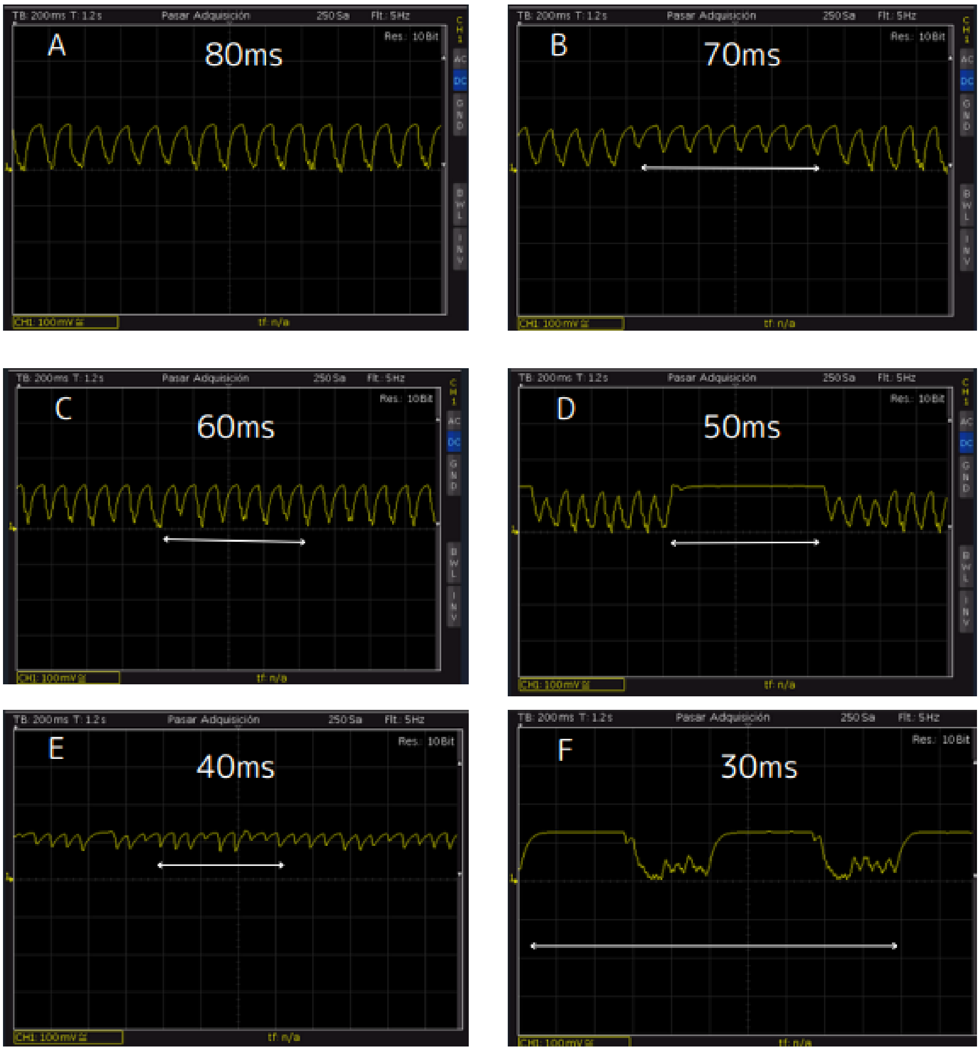

Figure S8. (a) Measurement of the minimum reliable light shutter interval (80 ms) for consistent beam modulation. (b-f) Oscilloscope readings showing irregularities in beam obstruction at shorter switching intervals (70–30 ms), including incomplete or unstable shutter movements. Experiments were conducted using a Rohde & Schwarz HMO1002 Series oscilloscope, a 10 mm photodiode, and a 10 mm light beam.

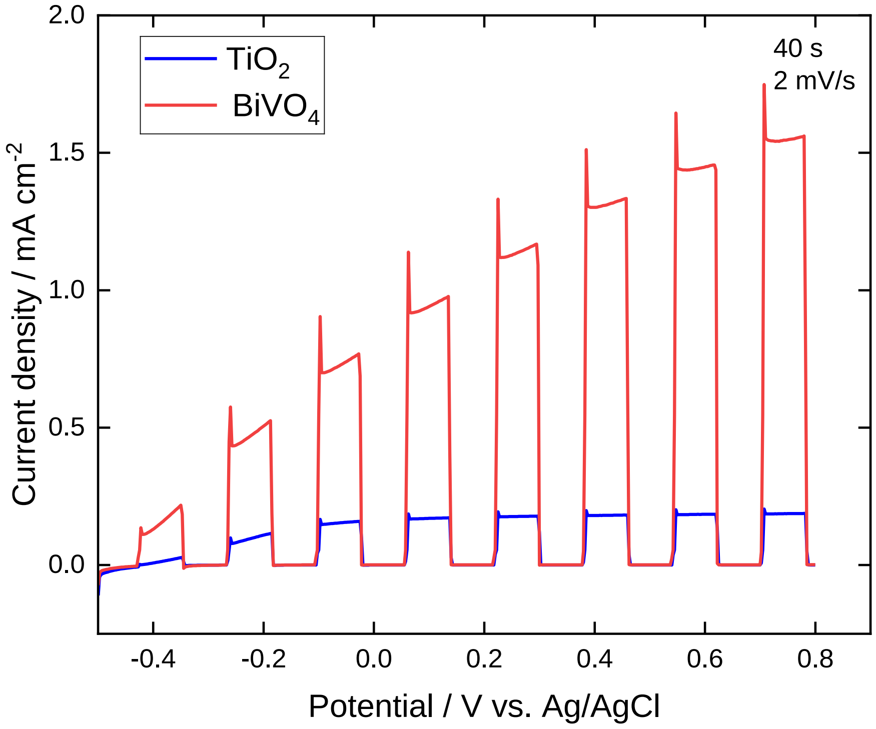

Figure S9. Linear sweep voltammograms of $BiVO_4$ and TiO_2 NTs photoelectrodes recorded in an H_3BO_3 buffer solution (pH 9.3) saturated with Ar at a scan rate of 2 mV s^{-1} conducted at 40 s light cut-off interval. The light intensity was set to 200 mW cm^{-2}. Potentials were recorded against an Ag/AgCl reference electrode, and light modulation timing was controlled by the Arduino-based light shutter system.

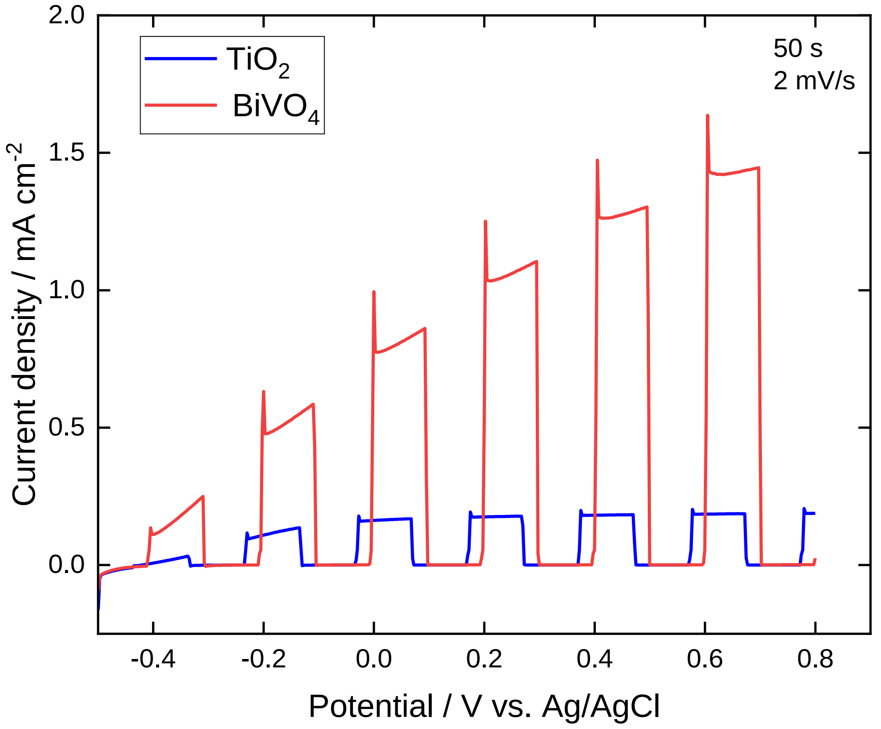

Figure S10. Linear sweep voltammograms of BiVO and TiO_2 NTs photoelectrodes recorded in an H_3BO_3 buffer solution (pH 9.3) saturated with Ar at a scan rate of 2 mV s^{-1} conducted at 50 s light cut-off interval. The light intensity was set to 200 mW cm^{-2}. Potentials were recorded against an Ag/AgCl reference electrode, and light modulation timing was controlled by the Arduino-based light shutter system.

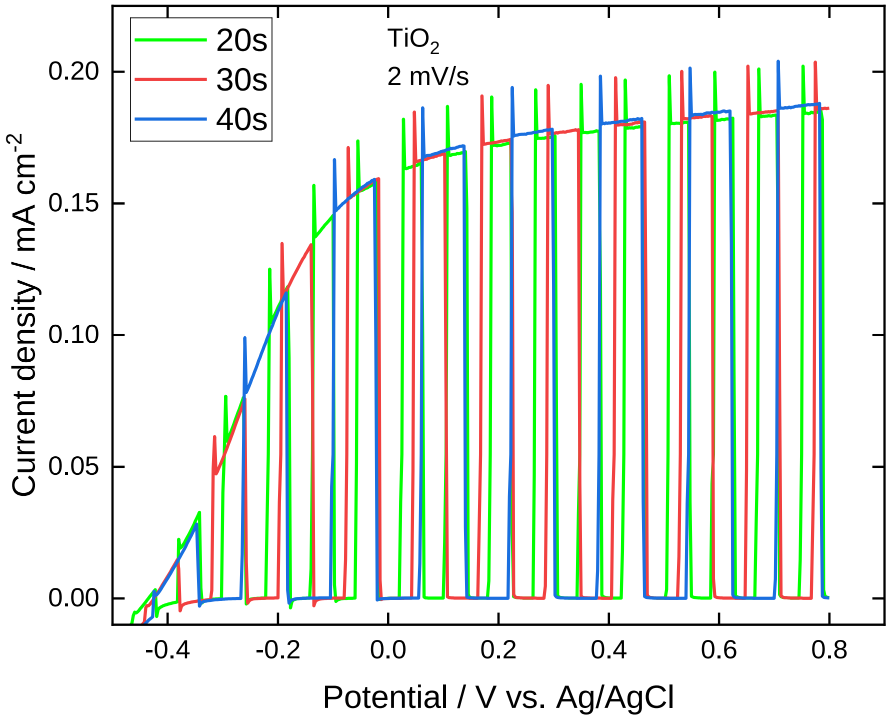

Figure S11. Linear sweep voltammograms of TiO_2 NTs photoelectrode recorded in an H_3BO_3 buffer solution (pH 9.3) saturated with Ar at a scan rate of 2 mV s^{-1}. Measurements were conducted with varying light cut-off intervals of 20, 30, 40 s. The light intensity was set to 200 mW cm^{-2}. Potentials were recorded against an Ag/AgCl reference electrode, and light modulation timing was controlled by the Arduino-based light shutter system.

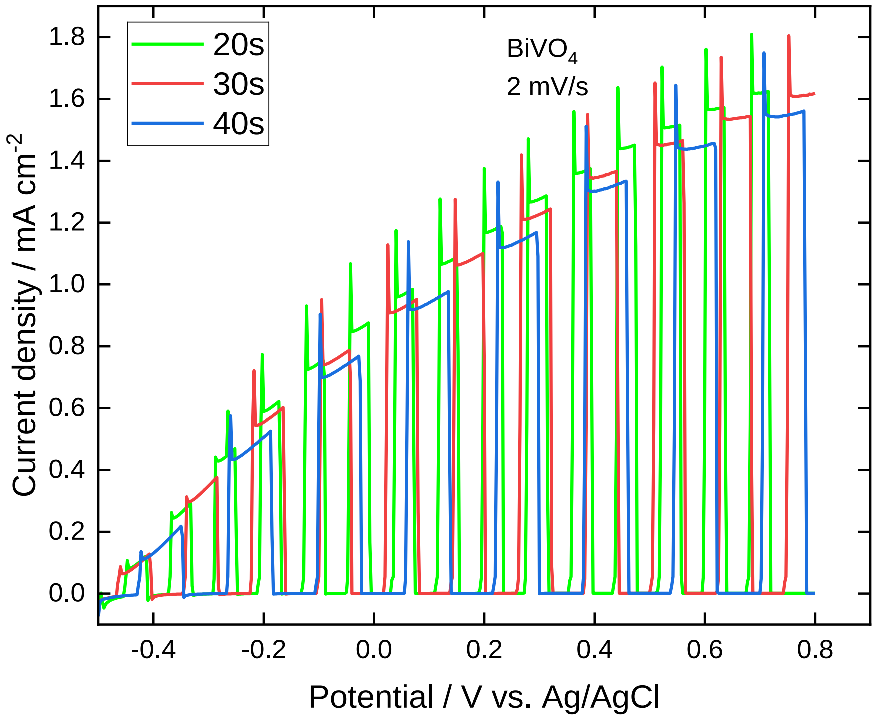

Figure S12. Linear sweep voltammograms of $BiVO_4$ photoelectrode recorded in an H_3BO_3 buffer solution (pH 9.3) saturated with Ar at a scan rate of 0.002 V s^{-1}. Measurements were conducted with varying light cut-off intervals of 20, 30, and 40 s. The light intensity was set to 200 mW cm^{-2}. Potentials were recorded against an Ag/AgCl reference electrode, and light modulation timing was controlled by the Arduino-based light shutter system.

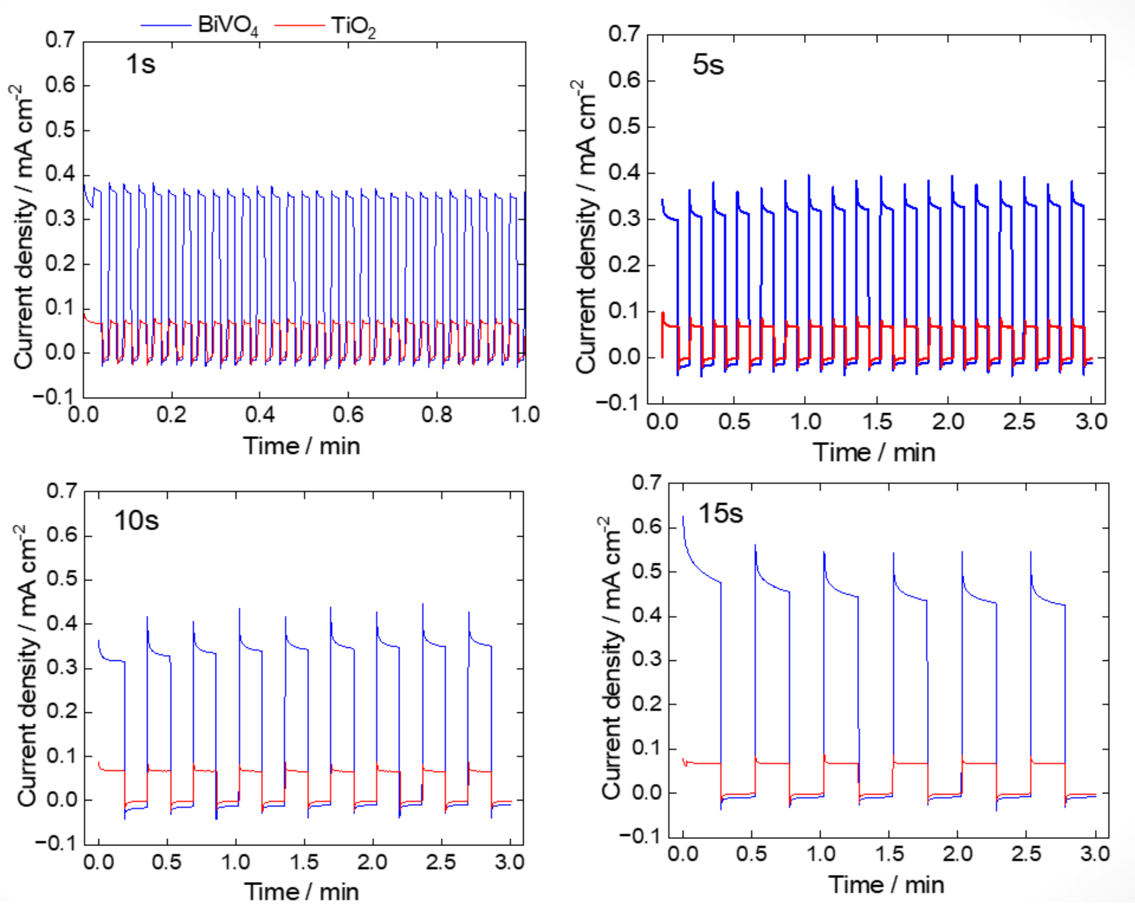

Figure S13. Chronoamperometries at 0.2 V *vs*. Ag/AgCl for BiVO and TiO_2 NTs photoelectrodes recorded in an H_3BO_3 buffer solution (pH 9.3) saturated with Ar for 3 min conducted at 1, 5, 10, and 15 s light cut-off interval. The light intensity was set to 200 mW cm^{-2}. The light exposure timing was controlled by the Arduino-based light shutter system.

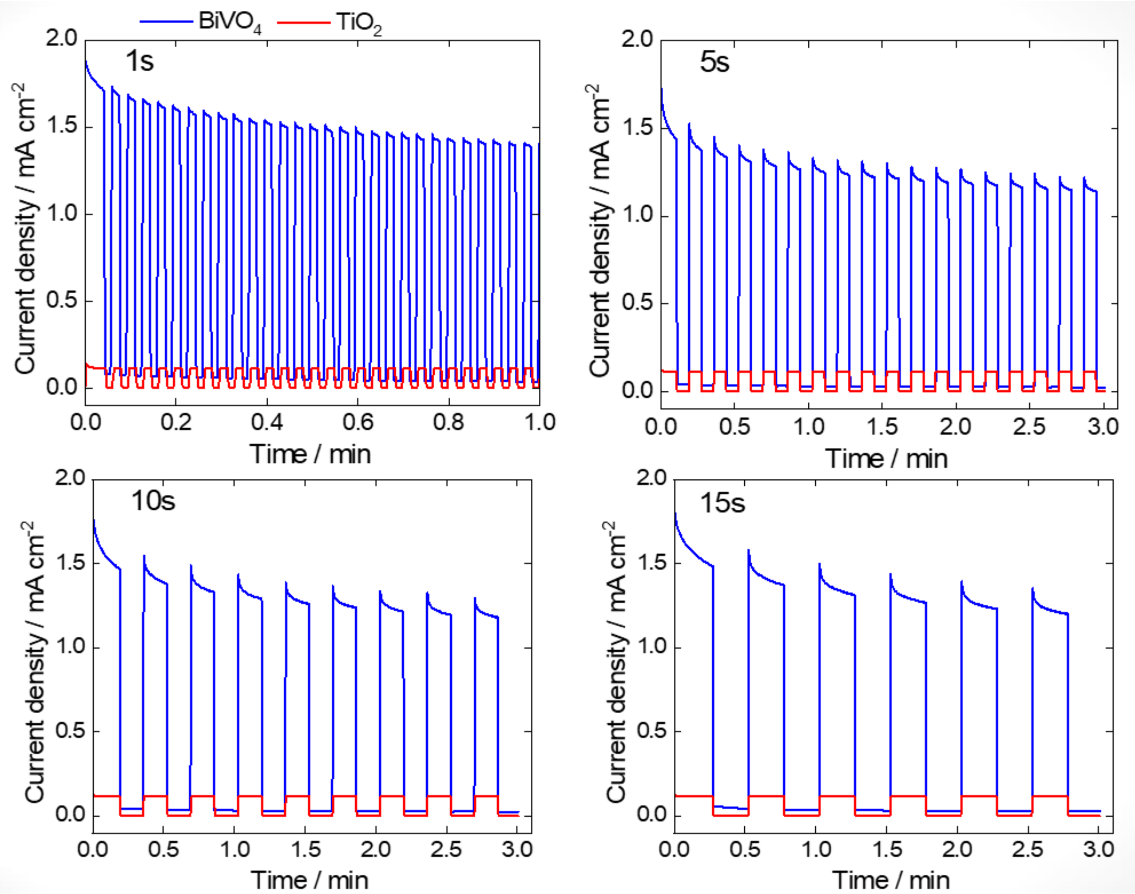

Figure S14. Chronoamperometries at 0.6 V *vs*. Ag/AgCl for BiVO and TiO_2 NTs photoelectrodes recorded in an H_3BO_3 buffer solution (pH 9.3) saturated with Ar for 3 min conducted at 1, 5, 10, and 15 s light cut-off interval. The light intensity was set to 200 mW cm^{-2}. The light exposure timing was controlled by the Arduino-based light shutter system.

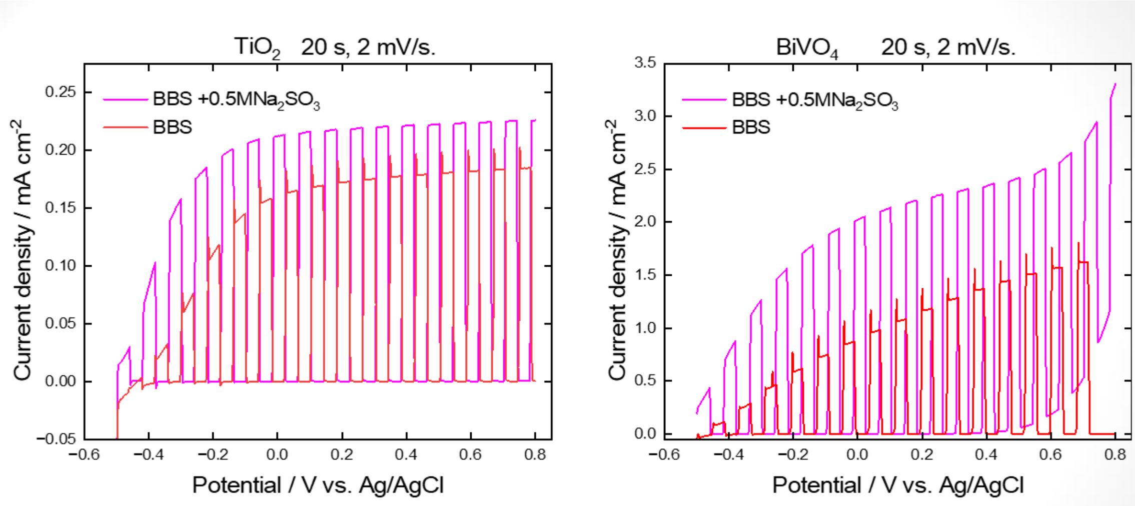

Figure S15. Linear sweep voltammograms of TiO_2 and $BiVO_4$ photoelectrode recorded in an H_3BO_3 buffer solution (pH 9.3) with and without 0.5 mol L^{-1} Na^{2}SO$_3$ saturated with Ar at a scan rate of 2 mV s^{-1}. Measurements were conducted with varying light cut-off intervals of 20 s. The light intensity was set to 200 mW cm^{-2}. Potentials were recorded against an Ag/AgCl reference electrode, and light switch timing was controlled by the Arduino-based light shutter system.

Table S1. Charge transfer efficiencies (η_{trans}) calculated using the formula $\eta_{trans} = \frac{J_{ss}}{J_{inst}}$ (expressed as a percentage), where J_{ss} is the steady-state current density, and J_{inst} is the instantaneous current density at the onset of the "light-on" state. Values calculated as an average spike of voltammograms at 2 mV s^{-1} from -0.2 to 0.2 V for 20, 25, 40, and 50 s of light cut-off intervals.

	TiO_2			$BiVO_4$		
Cut-off time(s)	J_{ss}	J_{inst}	η_{trans}	J_{ss}	J_{inst}	η_{trans}
20	0.149	0.171	87%	0.915	1.126	81%
25	0.146	0.17	86%	0.898	1.09	82%
40	0.145	0.168	86%	0.921	1.118	82%
50	0.153	0.177	86%	0.987	1.185	83%
